# Association of doctor-patient assistants on surgical efficiency and healthcare quality in gynecological ambulatory surgery: A retrospective study

**DOI:** 10.1097/MD.0000000000048091

**Published:** 2026-03-13

**Authors:** Liping Zhang, Fei Wang, Yuqin Liu, Yongting Zhao, Xue Bai, Jing Ma, Lijie Pan, Liehong Wang

**Affiliations:** a Department of Gynaecology and Obstetrics, Qinghai Red Cross Hospital, Xining, Qinghai Province, China; b College of Clinical Medicine, Qinghai University, Xining, Qinghai Province, China; c Nursing Department, Qinghai Red Cross Hospital, Xining, Qinghai Province, China.

**Keywords:** DPA, gynecological ambulatory surgery, nursing quality, patient satisfaction, surgical efficiency

## Abstract

This study aimed to explore the association between the implementation of doctor-patient assistants (DPA) and surgical efficiency as well as healthcare quality in gynecological ambulatory surgery. A retrospective cohort study was conducted on 157 patients undergoing gynecological ambulatory surgeries at Qinghai Red Cross Hospital between January 2024 and January 2025. The control group (n = 89) received conventional management, while the study group (n = 68) was managed with involvement of DPAs. Surgical efficiency, patients’ negative emotions, nursing quality scores, surgery cancelation rates, and patient satisfaction were evaluated. The study group showed a higher rate of complete preoperative preparation, shorter patient transfer time, reduced waiting time for the first surgery case, and shorter intervals between consecutive surgeries (*P* < .05). Negative emotion scores were significantly lower in the study group (*P* < .05). Nursing quality scores were higher in the study group (*P* < .05). The surgery cancelation rate was lower in the study group (*P* < .05). Patient satisfaction scores were significantly higher in the study group (*P* < .05). In this retrospective study, the involvement of DPA in gynecological ambulatory surgery workflows was associated with enhanced surgical efficiency and improved healthcare quality.

## 1. Introduction

Surgery remains a crucial therapeutic modality for various diseases. Traditionally, patients undergoing surgical procedures require prolonged hospital stays involving preoperative preparation, surgery, and postoperative care. Ambulatory surgery allows patients to complete admission, surgery, postoperative observation, and discharge within 24 hours.^[1]^ With ongoing advancements, ambulatory surgery has gradually been applied to select gynecological procedures such as benign tumor removals, endoscopic surgeries, and family planning surgeries, characterized by clear diagnosis, low surgical risk, and rapid postoperative recovery.^[2]^ Ambulatory surgery optimizes healthcare resource utilization and increases bed turnover rates, alleviating bed shortages, while receiving high acceptance from patients.^[3]^ However, increasing ambulatory surgery volumes have revealed management inconsistencies and workflow challenges that adversely affect surgical efficiency and nursing quality.^[4]^ Therefore, effective interventions are essential. DPA serve as vital coordinators linking patients, healthcare providers, and workflow processes.^[5]^ By enhancing communication efficiency, streamlining process management, and strengthening nursing care, they contribute to improved surgical efficiency and healthcare quality.^[6]^ This study explores the effects of instituting DPA on key metrics including surgical efficiency, patient anxiety and depression, nursing quality, and patient satisfaction in gynecological ambulatory surgery.

## 2. Materials and methods

### 2.1. General information

A retrospective analysis was performed on 157 gynecological ambulatory surgery patients treated at Qinghai Red Cross Hospital from January 2024 to January 2025. The control group (n = 89) received conventional management, and the study group (n = 68) was managed under DPA. The research group and the control group were conducted simultaneously. The research group was from January 2024 to January 2025, while the control group was also from January 2024 to January 2025. After the patient was admitted to the hospital, the attending doctor provided detailed explanations to the patient about the potential benefits and risks of the 2 modes. The patient then thought deeply and chose the appropriate mode based on their own situation, and signed the informed consent form. Baseline characteristics were collected for both groups. This retrospective study adhered to the principles of the Helsinki Declaration and received approval from the Ethics Committee of Qinghai Red Cross Hospital (LW-2025-84).

### 2.2. Inclusion and exclusion

Criteria Inclusion criteria: meeting indications for ambulatory surgery; complete clinical data available; normal communication ability; the ethical committee of Qinghai Red Cross Hospital has approved this study, and the participants have signed written informed consent forms, agreeing to participate in this research. Exclusion criteria: contraindications to surgery or anesthesia; (2) refusal of ambulatory surgery after preoperative communication; presence of other internal or surgical comorbidities.

### 2.3. Intervention methods

Control group: conventional management including preoperative assessment of patient condition and psychological status, assistance with required examinations, and preparation including dietary, skin preparation, hygiene, and medication protocols. Patients were informed about surgical cooperation and precautions. Postoperative care involved close monitoring of vital signs and condition changes, targeted nursing interventions, and guidance on diet and lifestyle. Discharge instructions and follow-up schedules were provided upon meeting discharge criteria.

**Figure 1.**
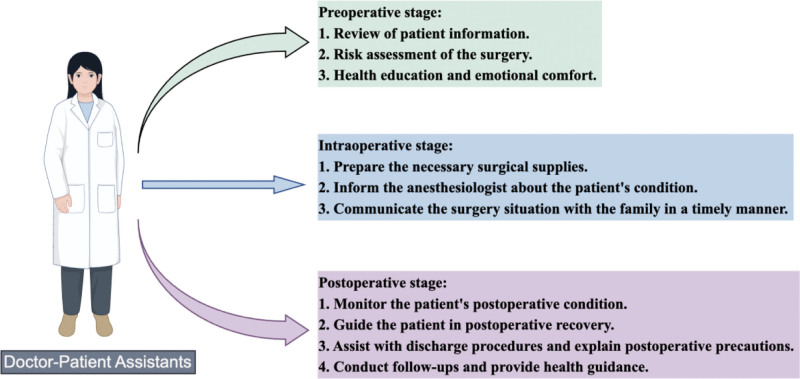
The workflow of the DPA. DPA=doctor-patient assistants.

Study group: surgical workflow was managed by a DPA, a qualified nurse with extensive experience. The DPAs were registered nurses with a minimum of 5 years of experience in gynecological surgery, who underwent a standardized 4-week training program covering patient coordination, risk assessment, enhanced communication techniques, and ambulatory surgery protocols (see Fig. 1). Preoperative phase – Data review: the DPA preemptively checked patient records, notified patients to complete any missing examinations, and coordinated with operating room administrators to schedule surgeries based on patient conditions, prioritizing critical cases to enhance turnover efficiency. Risk assessment: initial evaluation of anesthetic contraindications and intraoperative bleeding risk; multidisciplinary consultation arranged for patients with comorbidities to confirm ambulatory surgery suitability. Health education: disease-related knowledge, surgery significance, and procedures were communicated face-to-face and via health handbooks to improve patient awareness. The DPA reconfirmed preoperative preparations 1 day before surgery and assisted patient admission and emotional support on surgery day, verifying patient information and status with nursing staff to ensure timely surgery commencement. Intraoperative phase: surgical consumables were prepared in advance according to surgery type to avoid delays; equipment performance was verified on surgery day. The DPA communicated patient assessment results with anesthesiologists and operating room nurses to facilitate smooth procedures. Progress updates were provided to family members every 30 minutes, and the DPA assisted surgeons in timely communication with family in case of intraoperative emergencies such as excessive bleeding requiring transfusion. Postoperative phase: monitoring of vital signs, pain, and vaginal bleeding was conducted, with timely reporting to doctors. The DPA guided rehabilitation exercises; upon meeting discharge criteria via the Ambulatory Surgery Patient Discharge Assessment Form, assisted with discharge procedures and provided post-discharge instructions. Follow-up was conducted on days 1, 3, 7, and 14 to assess recovery and provide health guidance.

### 2.4. Outcome measures

Negative emotion was evaluated by the self-rating anxiety scale (SAS) and self-rating depression scale (SDS). Scores ≥50 on SAS and ≥53 on SDS indicated anxiety and depression symptoms, with higher scores correlating with severity. Nursing quality was measured by a nursing quality questionnaire scored out of 10, with higher scores indicating better quality. Patient satisfaction was assessed using a service satisfaction questionnaire (an institutional tool) covering domains such as preoperative preparation time, intraoperative comfort, information perception, and service attitude. Total scores range from 0 to 100.

### 2.5. Statistical analysis

Data were analyzed using SPSS 23.0 (IBM Corporation, New York). Continuous variables are expressed as mean ± standard deviation and were compared by *t*-test; results are reported with *P*-values and 95% confidence intervals for the mean differences. Categorical variables as n (%) and compared by chi-square test. A Bonferroni correction was applied to adjust for multiple comparisons, with the significance threshold set at *P* < .05/n (where n is the number of pairwise comparisons). Statistical significance for single comparisons was set at *P* < .05.

## 3. Results

### 3.1. Baseline characteristics

No significant differences were observed between groups regarding age, surgery type, educational level, and living area (*P* > .05), as shown in Table 1.

**Table 1:** Comparison of baseline characteristics.

Group	Control group (n = 89)	Study group (n = 68)	*χ*^2^/*t*	*P* value
Age (yr)	39.54 ± 3.25	40.04 ± 3.18	0.964	.336
Type of surgery				
Transcervical resection of myoma	29 (32.58)	22 (32.35)	0.001	.976
Transcervical resection of polyp	27 (30.34)	20 (29.41)		
Transcervical resection of septum	22 (24.72)	17 (25.00)		
Others	11 (12.36)	9 (13.24)		
Educational level				
≤Junior high school	69 (77.53)	55 (80.88)	0.261	.609
≥High school	20 (22.47)	13 (19.12)		
Residence				
City	66 (74.16)	50 (73.53)	0.008	.929
Countryside	23 (25.84)	18 (26.47)		

### 3.2. Surgical efficiency

The study group demonstrated higher rates of complete preoperative preparation, shorter times for patient transfer, waiting for the first surgery, and interval between consecutive surgeries compared with the control group (*P* < .05), as detailed in Table 2.

**Table 2:** Comparison of surgical efficiency.

Group	Control group (n = 89)	Study group (n = 68)	Mean difference (95% CI)	*χ*^2^/*t*	*P* value
Complete preoperative preparation	73 (82.02)	64 (94.12)	–	5.073	.024
Times for patient transfer (min)	41.63 ± 1.56	23.74 ± 3.04	17.89 (7.56–28.36)	47.903	<.001
Waiting for the first surgery (min)	71.25 ± 3.32	45.46 ± 4.13	25.79 (11.53–39.26)	43.370	<.001
Interval between consecutive surgeries (min)	36.34 ± 3.34	22.43 ± 3.17	13.91 (8.32–20.11)	26.430	<.001

CI = confidence interval.

### 3.3. Negative emotion scores

Postoperative SAS and SDS scores in the study group were significantly lower than in the control group (*P* < .05). Both groups showed reductions in anxiety and depression scores compared to preoperative levels (*P* < .05), as shown in Table 3.

**Table 3:** Comparison of negative emotion scores.

Group	Preoperation		Mean difference (95% CI)	*t*	*P* value	Postoperation		Mean difference (95% CI)	*t*	*P* value
	Control group (n = 89)	Study group (n = 68)				Control group (n = 89)	Study group (n = 68)			
SAS	58.72 ± 1.85	58.64 ± 1.71	0.08 (0.01–1.09)	0.277	.782	43.40 ± 1.53*	38.49 ± 1.79*	4.91 (2.21–7.34)	18.504	<.001
SDS	60.56 ± 1.88	60.27 ± 2.08	0.29 (0.17–0.33)	0.902	.368	44.06 ± 1.48*	39.38 ± 1.64*	4.68 (1.89–7.01)	18.732	<.001

Compared with the preoperative state of the same group.

CI = confidence interval, SAS = self-rating anxiety scale, SDS = self-rating depression scale.

* *P* < .05.

### 3.4. Nursing quality scores

Nursing quality scores were significantly higher in the study group compared to the control group (*P* < .05), as presented in Table 4.

**Table 4:** Comparison of nursing quality scores.

Group	Control group (n = 89)	Study group (n = 68)	Mean difference (95% CI)	*t*	*P* value
Preparing an operation	8.06 ± 0.55	9.41 ± 0.18	−1.35 (−1.89 to −1.01)	5.042	<.001
Operation environment	8.12 ± 0.42	9.50 ± 0.16	−1.38 (−1.73 to −0.88)	7.075	<.001
Sanitary disinfection	8.10 ± 0.52	9.51 ± 0.19	−1.41 (−1.94 to −1.08)	6.190	<.001
Instrument management	8.04 ± 0.34	9.41 ± 0.26	−1.37 (−1.91 to −0.92)	7.459	<.001
Nursing record	8.13 ± 0.33	9.44 ± 0.22	−1.31 (−1.81 to −1.04)	6.691	<.001
Operation safety	8.19 ± 0.41	9.62 ± 0.13	−1.43 (−2.09 to −0.77)	8.329	<.001
Surgical operation	8.11 ± 0.35	9.46 ± 0.11	−1.35 (−1.93 to −0.89)	7.947	<.001

CI = confidence interval.

### 3.5. Surgery cancellation rates

The study group had a significantly lower surgery cancelation rate than the control group (*P* < .05), as presented in Table 5.

**Table 5:** Comparison of surgery cancellation rates.

Reason for cancellation	Control group (n = 89)	Study group (n = 68)	Rate difference (95% CI)	*χ* ^2^	*P* value
Preoperative discomfort	6 (6.74)	2 (2.94)	–	–	–
Incomplete preoperative examination	4 (4.49)	1 (1.47)	–	–	–
Time conflict	4 (4.49)	1 (1.47)	–	–	–
Patient’s personal factors	5 (5.62)	2 (2.94)	–	–	–
Total cancellations	19 (21.34)	6 (8.82)	12.52 (1.51–23.53)	4.517	.034

CI = confidence interval.

### 3.6. Patient satisfaction

Patient satisfaction scores were significantly higher in the study group than in the control group (*P* < .05), as shown in Table 6.

**Table 6:** Comparison of patient satisfaction.

Group	Control group (n = 89)	Study group (n = 68)	Rate difference (95% CI)	*χ* ^2^	*P* value
Very satisfied	38 (42.70)	44 (64.71)	–	–	–
Slightly satisfied	36 (40.45)	21 (30.88)	–	–	–
Not satisfied	15 (16.85)	3 (4.41)	–	–	–
Satisfaction	74 (83.15)	65 (95.59)	−12.44 (−20.91 to −3.97)	5.879	.015

CI = confidence interval.

## 4. Discussion

At present, ambulatory surgery has been widely adopted due to its advantages of high efficiency and quality.^[7]^ Compared with traditional surgery, ambulatory surgery reduces patient hospitalization time, with admission to discharge typically within 24 hours.^[8]^ This short timeframe alleviates patients’ anxiety and panic associated with waiting for surgery, minimizes disruption to their daily life and work, and is especially suitable for individuals with academic or professional commitments or those requiring rapid postoperative recovery.^[9]^ Furthermore, the shortened hospital stay reduces patients’ exposure to hospital-acquired pathogens, thereby lowering the risk of cross-infection caused by prolonged contact with the hospital environment.^[10]^ Ambulatory surgery also enhances the utilization of medical resources, alleviating the shortage of inpatient beds, particularly in hospitals with high surgical volumes.^[11]^ It optimizes medical service efficiency, allows patients to quickly return to their daily life, reduces economic burden, and improves the overall patient experience.^[12]^ Despite increasing support and promotion of ambulatory surgery, practical implementation still faces challenges such as limited patient awareness and inadequate preoperative preparation, which hinder improvements in operational efficiency.^[13]^ To address these issues, hospitals have established the role of DPA – professionals possessing specialized knowledge and strong learning ability who thoroughly understand medical and administrative protocols and facilitate effective communication between patients and healthcare providers.^[14,15]^ DPA serve as a vital communication bridge and fully endorse the concept of planned surgical services, thereby providing essential support for high-quality surgical care^[16]^ (see Fig. 1).

In this study, the group managed with DPA demonstrated a higher rate of complete preoperative preparation, shorter patient transfer times, reduced waiting times for the first surgery, and decreased intervals between consecutive procedures. In conventional ambulatory surgery workflows, patients often experience delays of 2 to 3 days due to incomplete examinations or scheduling issues. With DPA, patient information was proactively verified and coordinated with operating room staff to assist patients in completing necessary tests and scheduling surgeries, effectively shortening preoperative preparation and waiting periods. Additionally, the use of risk assessment tools enabled timely exclusion of high-risk patients unsuitable for ambulatory surgery, preventing intraoperative cancelations due to exacerbated conditions. Preoperative communication with anesthesiologists and operating room nurses ensured timely surgery starts and confirmed the availability and functionality of surgical equipment and instruments, thereby improving operating room turnover efficiency. The study group also exhibited lower scores of negative emotions, likely attributable to the personalized communication strategies developed by DPA for different patients. These assistants provided early education on disease and surgical information, informed patients about required examinations and preparatory steps, and enhanced patients’ awareness of ambulatory surgery. Continuous accompaniment by DPA during hospitalization increased patients’ trust and sense of security. The shortened hospital stay further mitigated anxiety associated with prolonged hospitalization, facilitating earlier discharge and better psychological adjustment, effectively alleviating negative emotions. Nursing quality scores were higher in the study group, reflecting strengthened patient safety management following doctor-patient assistant intervention. Preoperative risk assessments, dynamic monitoring of examination results, and implementation of a tripartite verification mechanism among anesthesiologists, surgical nurses, and DPA ensured readiness of surgical equipment and materials. Postoperative monitoring enabled early identification of complications and prompt reporting to physicians for targeted treatment. Discharge guidance and regular follow-up contributed to overall nursing quality improvement. The rate of surgery cancelations was lower in the study group. Reasons for cancelations in ambulatory surgery include patient-related factors (inadequate preparation or condition changes), resource-related factors (operating room conflicts or poor staff coordination),and process-related factors (delayed information transfer, unreasonable scheduling). DPA mitigated these factors through meticulous patient management, precise information transmission, and efficient resource coordination, thereby reducing cancelation rates. Experienced DPA managing ambulatory surgery shortened patient waiting times, standardized preoperative evaluation and surgical scheduling, and optimized postoperative management processes, collectively improving overall diagnostic and treatment efficiency. Effective preoperative communication and preparation ensured smooth surgical procedures, while close postoperative monitoring and health guidance guaranteed clinical safety and enhanced nursing experience, ultimately elevating patient satisfaction.

### 4.1. Limitations

This study has several limitations that should be considered when interpreting the results. First, due to its retrospective and non-randomized design, the study is susceptible to selection bias and unmeasured confounding factors, despite statistical adjustments aimed at minimizing these risks. Specifically, the concurrent but non-randomized allocation based on patient preference, while reflecting real-world clinical practice, may introduce systematic differences between groups that could affect outcomes. Second, the single-center nature of our investigation may restrict the generalizability of the findings to other settings or populations with different demographic or clinical characteristics. Third, the assessment of nursing quality and patient satisfaction relied on institution-specific tools that have not been formally validated, which may affect the reproducibility and comparability of these metrics. Finally, the absence of blinding in the intervention and evaluation phases introduces the potential for observer bias. Future prospective, multi-center studies utilizing validated instruments and blinded assessment procedures are warranted to confirm and extend our findings.

## 5. Conclusion

In this retrospective study, the involvement of DPA in the ambulatory surgery process for gynecological procedures was associated with optimized medical service quality, improved surgical efficiency, alleviation of patient psychological burden, reduced cancelation rates, and higher patient satisfaction. These findings suggest that this approach may be worthy of broader clinical application and evaluation. Future prospective, randomized controlled trials are warranted to confirm these observed associations and to explore the cost-effectiveness of the DPA model.

## Acknowledgments

Fei Wang and Liping Zhang contributed to this article equally. Lijie Pan and Liehong Wang contributed to this article equally. Thank all the members of the Gynecology Department Grade 4 of Qinghai Red Cross Hospital.

## Author contributions

**Conceptualization:** Liping Zhang, Fei Wang, Yuqin Liu, Yongting Zhao, Xue Bai, Jing Ma, Lijie Pan, Liehong Wang.

**Data curation:** Liping Zhang, Fei Wang, Yuqin Liu, Yongting Zhao, Xue Bai, Jing Ma, Lijie Pan.

**Formal analysis:** Liping Zhang, Fei Wang, Yuqin Liu, Yongting Zhao, Jing Ma.

**Funding acquisition:** Liping Zhang, Fei Wang.

**Investigation:** Liping Zhang, Fei Wang, Yuqin Liu, Xue Bai, Lijie Pan, Liehong Wang.

**Methodology:** Liping Zhang, Fei Wang, Yuqin Liu, Xue Bai, Jing Ma, Lijie Pan.

**Project administration:** Liping Zhang, Fei Wang, Jing Ma.

**Resources:** Liping Zhang, Fei Wang.

**Software:** Liping Zhang, Fei Wang, Lijie Pan, Liehong Wang.

**Supervision:** Liping Zhang, Fei Wang, Jing Ma, Lijie Pan, Liehong Wang.

**Validation:** Liping Zhang, Fei Wang, Liehong Wang.

**Visualization:** Liping Zhang, Fei Wang, Xue Bai, Jing Ma, Lijie Pan, Liehong Wang.

**Writing – original draft:** Liping Zhang, Fei Wang, Yuqin Liu, Xue Bai, Lijie Pan, Liehong Wang.

**Writing – review & editing:** Liping Zhang, Fei Wang, Yuqin Liu, Xue Bai, Jing Ma, Lijie Pan, Liehong Wang.
